# A Genetic Study of *Nif*-Associated Genes in a Hyperthermophilic Methanogen

**DOI:** 10.1128/spectrum.02093-21

**Published:** 2022-02-02

**Authors:** Thomas J. Lie, Yang P. Kuo, Mara Leite, Kyle C. Costa, Caroline S. Harwood, John A. Leigh

**Affiliations:** a Department of Microbiology, University of Washington, Seattle, Washington, USA; Technical University of Denmark

**Keywords:** *Methanocaldococcus*, genetics, nitrogen fixation

## Abstract

*Methanocaldococcus* sp. strain FS406-22, a hyperthermophilic methanogen, fixes nitrogen with a minimal set of known *nif* genes. Only four structural *nif* genes, *nifH*, *nifD*, *nifK*, and *nifE,* are present in a cluster, and a *nifB* homolog is present elsewhere in the genome. *nifN*, essential for the final synthesis of the iron-molybdenum cofactor of nitrogenase in well-characterized diazotrophs, is absent from FS406-22. In addition, FS406-22 encodes four novel hypothetical proteins, and a ferredoxin, in the *nif* cluster. Here, we develop a set of genetic tools for FS406-22 and test the functionality of genes in the *nif* cluster by making markerless in-frame deletion mutations. Deletion of the gene for one hypothetical protein, designated Hp4, delayed the initiation of diazotrophic growth and decreased the growth rate, an effect we confirmed by genetic complementation. NifE also appeared to play a role in diazotrophic growth, and the encoding of Hp4 and NifE in a single operon suggested they may work together in some way in the synthesis of the nitrogenase cofactor. No role could be discerned for any of the other hypothetical proteins, nor for the ferredoxin, despite the presence of these genes in a variety of related organisms. Possible pathways and evolutionary scenarios for the synthesis of the nitrogenase cofactor in an organism that lacks *nifN* are discussed.

**IMPORTANCE** *Methanocaldococcus* has been considered a model genus, but genetic tools have not been forthcoming until recently. Here, we develop and illustrate the utility of positive selection with either of two selective agents (simvastatin and neomycin), negative selection, generation of markerless in-frame deletion mutations, and genetic complementation. These genetic tools should be useful for a variety of related species. We address the question of the minimal set of *nif* genes, which has implications for how nitrogen fixation evolved.

## INTRODUCTION

Biological nitrogen fixation, the conversion of N_2_ to NH_3_ by organisms, is the major source of biologically available nitrogen for life on earth. The process is widespread among Bacteria and also occurs in a variety of methanogenic Archaea. Early studies in Bacteria identified a large number of genes specific to nitrogen fixation (for example, 20 *nif* genes in Klebsiella pneumonia, [1]), and it has been suggested that the origin of a large number of *nif* genes coincided with the acquisition of nitrogen fixation by aerobes (2). In contrast, recent examination of genomes of putative nitrogen fixers suggests that the minimal set of *nif* genes is much smaller (3–6). This minimal set may include *nifD* and *nifK* encoding the nitrogenase enzyme which catalyzes the fixation of N_2_ to NH_3_, *nifH* encoding nitrogenase reductase, and *nifE*, *nifN*, and *nifB* encoding proteins involved in the synthesis of the iron-molybdenum cofactor (FeMoco) of nitrogenase (7). In the mesophilic methanogen Methanococcus maripaludis we identified a single *nif* operon containing *nifH*, *nifD*, *nifK*, *nifE*, *nifN*, and *nifX*, as well as *nifI_1_* and *nifI_2_* encoding proteins involved in negative regulation of nitrogenase activity (8–10). *nifB* was also present elsewhere in the genome. However, the minimal set of genes required for nitrogen fixation may be even fewer, as certain potential diazotrophs lack *nifN* and a few lack both *nifE* and *nifN* (3). Phylogenetic analysis has led to the suggestion that a nitrogenase with a FeMoco or a “proto-Mo-cofactor” in an organism lacking *nifN* or even lacking both *nifE* and *nifN* is ancestral (6).

**FIG 1:**
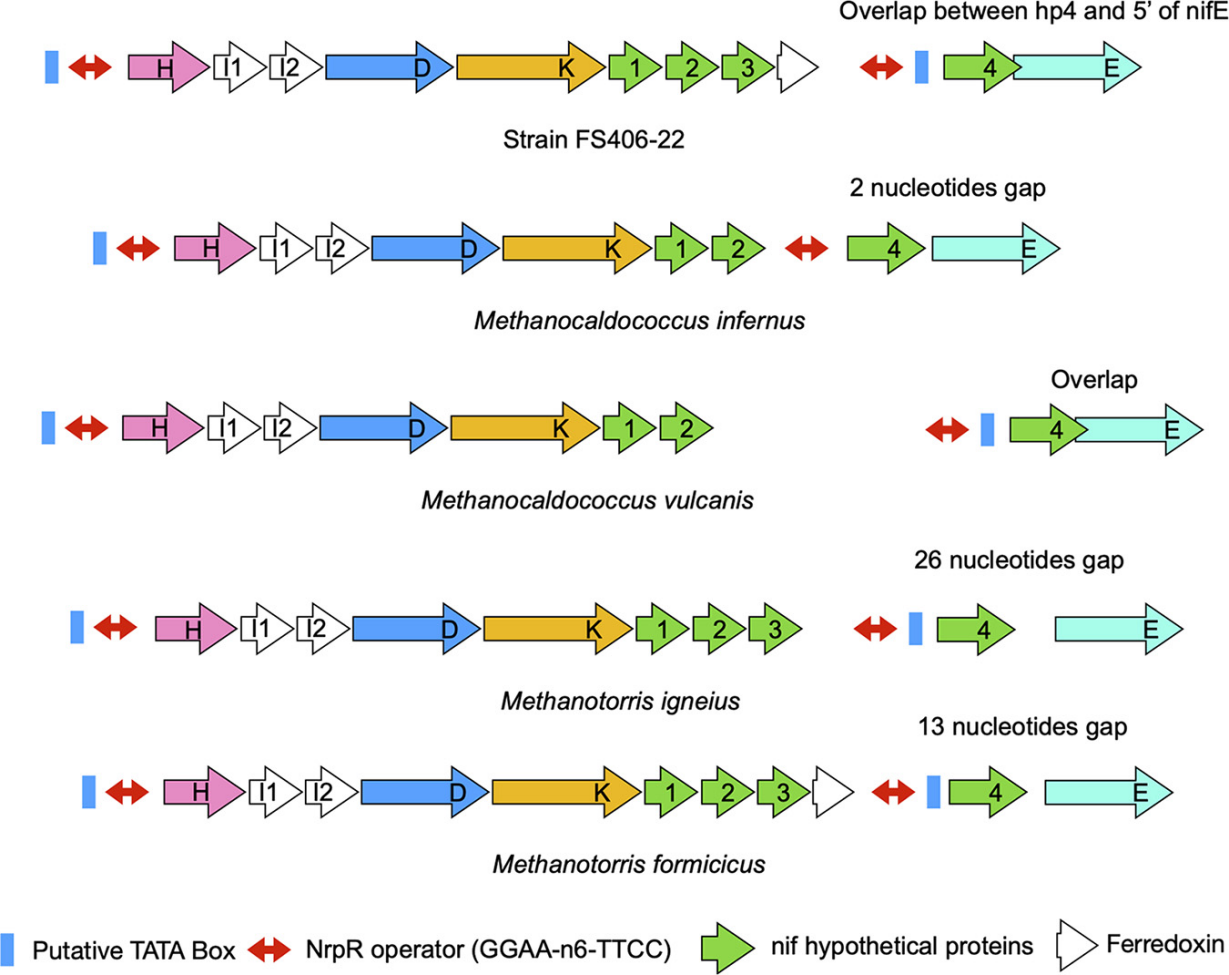
*nif* operon structures of hyperthermophilic methanogens. Shown are all diazotrophs known that contain *hp4*. The NrpR operator is a conserved binding site for the nitrogen regulator NrpR (12, 13). *nifI_1_* and *nifI_2_* encode proteins involved in negative regulation of nitrogenase activity (9, 10).

In this study, we focus on a species of hyperthermophilic methanogen, *Methanocaldococcus* strain FS406-22 (henceforth called FS406), which is capable of diazotrophic growth as high as 92°C, the highest temperature yet reported for biological nitrogen fixation (11). Representative of several species of diazotrophic thermophilic methanogens (Fig. 1), FS406 has two distinctive features. First, FS406 contains only five known *nif* genes (other than the regulatory *nifI_1_* and *nifI_2_*), yet is clearly capable of nitrogen fixation (11, this work). *nifH*, *nifD*, *nifK*, and *nifE* are present in a cluster (Fig. 1), and a *nifB* homolog is present elsewhere in the genome. Neither *nifN*, nor any other *nif* genes, are present anywhere in the genome. Second, FS406 contains four novel hypothetical proteins in its *nif* gene cluster, three following *nifK* in an apparent operon, and a fourth preceding *nifE* in an adjacent apparent operon (Fig. 1). Genes we have designated *hp1*, *hp2*, and *hp4* encode proteins with homologs only in *nif* clusters of thermophilic methanogens and have no detectable motifs or homologies that would indicate function. *hp3* has homologs in Archaea and Bacteria and contains similarity to anaerobic ribonucleoside triphosphate reductase with an ATP cone domain. While the hypothetical proteins have no homology to known nitrogen fixation proteins, their presence in apparent operons with known *nif* genes suggests they function in some way during nitrogen fixation. Furthermore, each apparent operon is preceded by a canonical TATA box and a specific operator sequence known to bind NrpR, a repressor of nitrogen assimilation genes in methanogens (12, 13). FS406 and some other thermophilic methanogens also contain a ferredoxin-encoding gene in their *nif* gene clusters.

Our goals in this study were 3-fold. First, we wanted to determine the possible importance for nitrogen fixation of the hypothetical proteins and the ferredoxin-encoding gene in the *nif* gene cluster in FS406. Second, we wanted to gain insight into the biosynthesis of the nitrogenase cofactor by examining the roles of *nifE* and the adjacent *hp4*. Third, we wanted to demonstrate the utility of genetic approaches in hyperthermophilic methanogens. While genetic tools have been used in other (nonmethanogenic) thermophilic Archaea (14), and we and others have used genetic tools in mesophilic methanogens, these tools have only recently been applied to thermophilic methanogens (15).

## RESULTS

### Development of a genetic system and generation of a *nifD* mutant (ML111).

To make mutations in FS406, we developed a “pop in-pop out” strategy as has been used in *M. maripaludis* and other organisms (14, 16). In this strategy, illustrated in Fig. S1, first positive selection is used to introduce a mutant gene into the organism. The mutant allele replaces the wild type by double homologous recombination (selectable maker inserted into the mutant gene), or the entire construct integrates into the genome by single homologous recombination forming a merodiploid (selectable marker in the vector). This is followed by a second step, where negative selection is used to replace or remove the integrated construct or vector, generating mutants containing only the mutant allele of the gene.

We first asked whether FS406 could be transformed using a polyethylene glycol (PEG) -based method similar to that used for *M. maripaludis* (17), and whether the same negative selection strategy would work (16). We constructed an in-frame deletion of the FS406 hypoxanthine phosphoribosyltransferase gene (*hpt*) and introduced it into the wild-type strain, selecting for resistance to the toxic analog 8-azahypoxanthine. The deletion mutant was obtained by double homologous recombination, and designated strain ML100. In this background *hpt* can be reintroduced together with a desired genetic construct in the first step described above, and negative selection using 8-azahypoxanthine can then be used in the second step.

**FIG 2:**
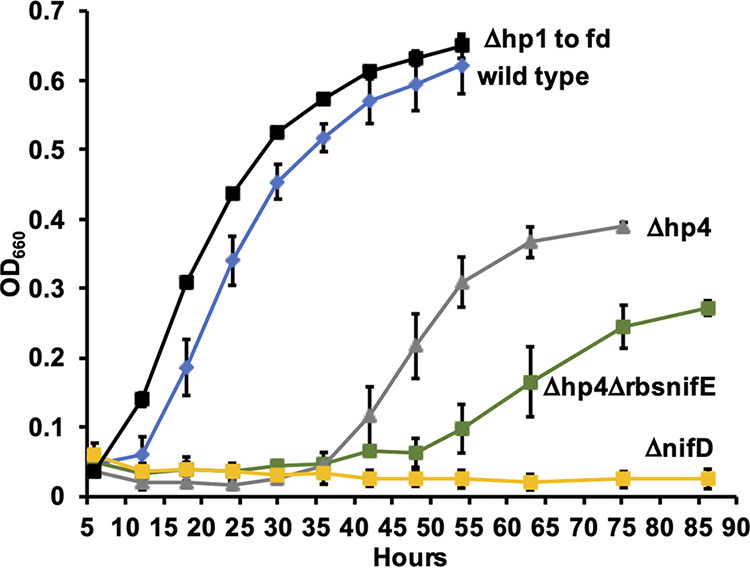
Diazotrophic growth of strains of FS406 at 75°C. Averages of three cultures and standard deviations are shown.

To test our genetic system, we generated a mutation of *nifD*, using the method shown in Fig. S1A. First we used neomycin selection to generate a strain in which an internal region of *nifD* was replaced with a cassette containing both a gene for aminoglycoside nucleotidyltransferase (*cohtk*, see below, providing neomycin resistance) and the *hpt* gene. To provide suitable expression in FS406, both genes were driven by strong promoters from the related species Methanocaldococcus jannaschii (see Materials and Methods). *cohtk* (see Materials and Methods) is a codon-optimized (for FS406) gene encoding a mutant highly thermostable aminoglycoside nucleotidyltransferase (18). In the second step we transformed with DNA containing an in-frame *nifD* deletion and selected with 8-azahypoxanthine. The deletion replaced the previously inserted cassette resulting in a strain containing a markerless in-frame deletion of *nifD*. As expected, the Δ*nifD* mutant was unable to grow with N_2_ as the sole nitrogen source (Fig. 2). (The Δ*nifD* mutant, and other mutants generated in this study, grew similarly to ML100 on ammonia, or better [Fig. S2]. Better than wild type growth could be due to relief of unneeded background expression of the genes that occurs in the wild type.)

### Mutation of *hp1*, *hp2*, *hp3*, and *fd* (ML200).

*hp1*, *hp2*, *hp3*, and the ferredoxin gene are contiguous at the 3′ end of an apparent operon, enabling us to delete all four genes in a single strain. Similar to the first step to generate the *nifD* mutant, we replaced an internal region of the *hp1*-*hp2*-*hp3*-*fd* cluster with the *cohtk*-*hpt* cassette. In this case, rather than proceeding with the second step to obtain a markerless in-frame deletion, we left the insertion in place and determined the phenotype of the Δ*hp1*-*hp2*-*hp3*-*fd*::*cohtk*-*hpt* mutant. The mutation had no deleterious effect on diazotrophic growth, and even appeared to alleviate a lag before growth began (Fig. 2). These results indicate that the four genes play no positive role in nitrogen fixation under the conditions tested. (In fact, the alleviation of a lag suggests the genes have a negative effect in the wild type under our conditions, possibly due to unneeded expression of genes that likely encode abundant proteins.) In separate experiments, we obtained mutants with markerless in-frame deletions of *hp1* and *fd*. Both mutants exhibited near-wild-type diazotrophic growth (data not shown), confirming the phenotype for these two genes.

### Deletion and complementation of *hp4*.

**FIG 3:**
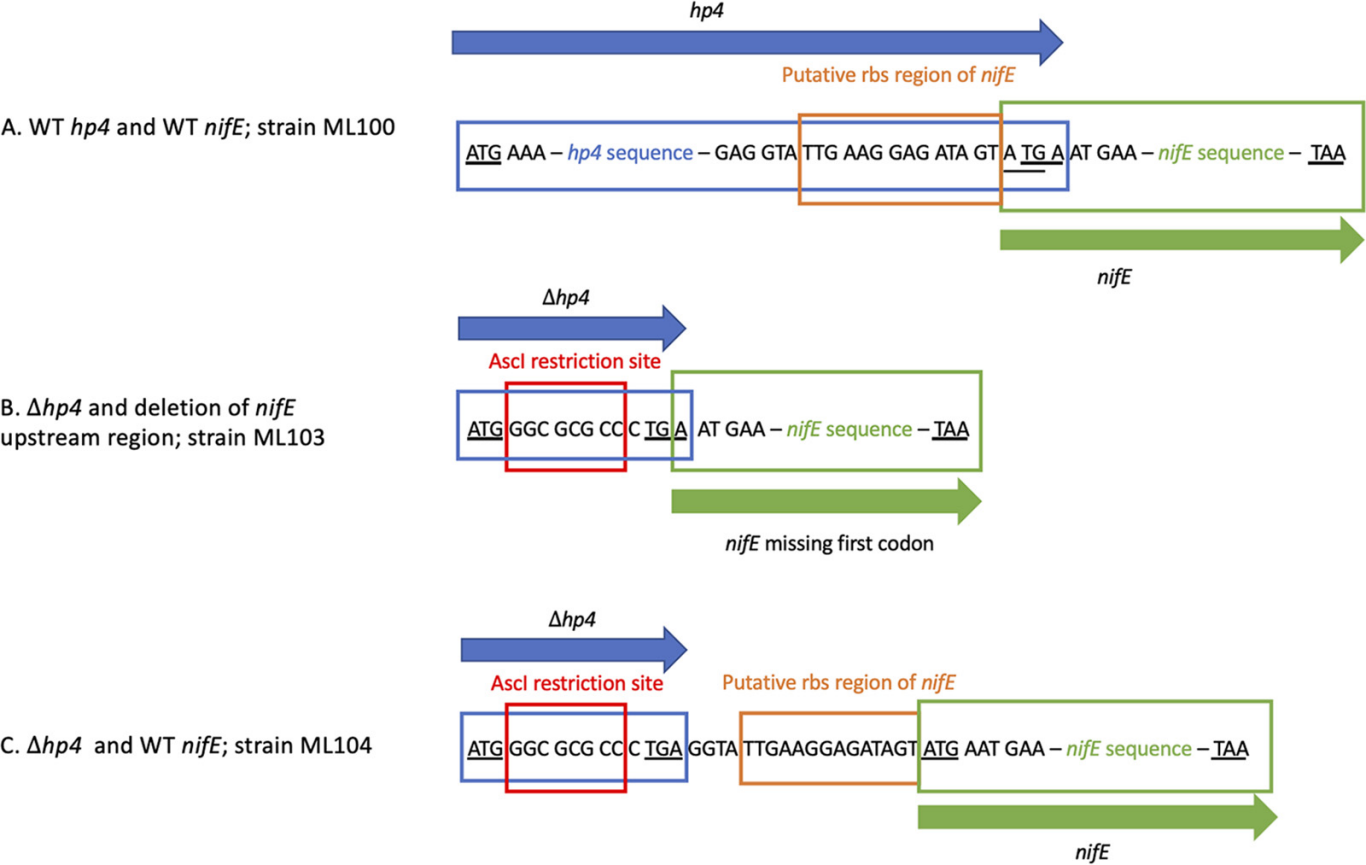
Structure of *hp4*-*nifE* region and deletion mutations.

**FIG 4:**
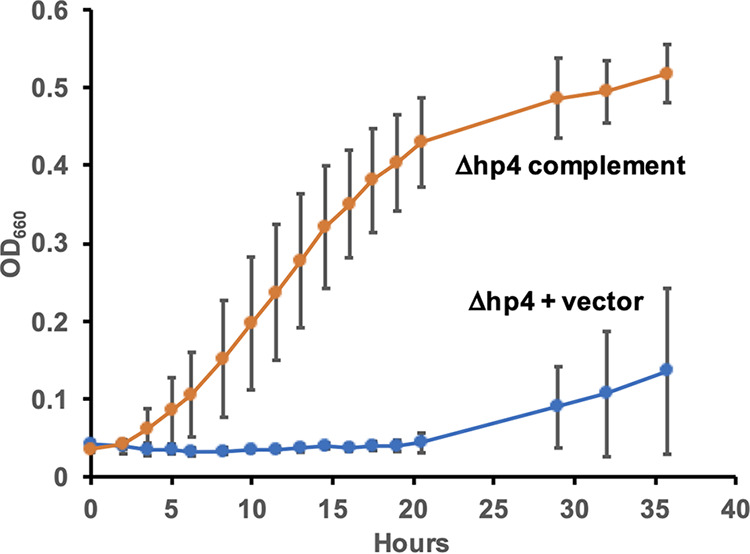
Complementation of Δ*hp4*. Diazotrophic growth at 75°C of Δ*hp4* strain with empty vector and Δ*hp4* strain with vector containing wild type *hp4*. Simvastatin selection was maintained throughout growth. Averages of four cultures and standard deviations are shown.

We next deleted *hp4*, using the method shown in Fig. S1B. *hp4* is upstream of *nifE* in an apparent two-gene operon, with a four-nucleotide overlap between the reading frames of the two genes (Fig. 1, Fig. 3A). We made two deletion strains. The first strain (Δ*hp4-*Δrbs*nifE*) contained an in-frame deletion of *hp4* and also eliminated the ribosome binding site and first codon of *nifE* (Fig. 3B). The second strain (Δ*hp4*) contained an in-frame deletion of *hp4* but left the ribosome binding site and reading frame of *nifE* intact (Fig. 3C). (The upstream region of *hp4*, including the ribosome binding site and the start codon, was left intact in both mutants.) Both strains were partially deficient in diazotrophic growth, exhibiting a lag and a decreased rate of growth (Fig. 2). (Both strains grew similarly to ML100 on ammonia, Fig. S2.) The Δ*hp4*Δrbs*nifE* mutant had a more severe phenotype than the Δ*hp4* mutant, suggesting that in addition to *hp4*, *nifE* may also play a positive role in nitrogen fixation. To test whether growth of the mutants could be due to the acquisition of a suppressor mutation, we inoculated fresh medium with diazotrophically grown cultures. Both strains retained a deficient phenotype (Fig. S3). To confirm the role of *hp4*, we then constructed and tested a strain in which Δ*hp4* was genetically complemented. For this purpose, we constructed an integration vector containing the *M. jannaschii* HMG-CoA (3-hydroxy-3-methyl-glutaryl-CoA) reductase gene, conferring simvastatin resistance and providing a second method in addition to neomycin resistance for positive selection. (HMG-CoA reductase is needed for lipid synthesis in Archaea and its overexpression can overcome its inhibition by statins [14, 15]). The vector also contained a locus from the FS406 genome as a neutral site for homologous recombination. Inserting wild type *hp4* into this vector and integrating it into the genome of the Δ*hp4* strain resulted in substantial enhancement of growth, compared to the Δ*hp4* strain with an empty vector (Fig. 4). These data suggest that *hp4* is not essential for nitrogen fixation but enhances the initiation and rate of nitrogen fixation.

## DISCUSSION

The development of genetic approaches in hyperthermophilic methanogens expands our tool set for investigations in these organisms. In the past, numerous studies have investigated the functions of proteins encoded in the genome of the model hyperthermophilic species Methanocaldococcus jannaschii (a nonnitrogen fixer). However, these studies have generally required expression of the genes in heterologous organisms. Only recently, a method for targeted mutagenesis in *M. jannaschii* was described (15). In that study, DNA segments were replaced by DNA containing the HMG-CoA reductase gene, but markerless in-frame deletions were not reported. Here, we have turned to another member of *Methanocaldococcus*, FS406, to investigate the role of genes in nitrogen fixation. We were able to transform FS406, implement both positive and negative selection, and obtain markerless in-frame deletions of several genes in the *nif* region. Two selectable agents proved useful for positive selection, neomycin (using either of two thermostable mutant versions of the *kat* gene encoding aminoglycoside nucleotidyltransferase), and simvastatin (using the *hmgA* gene encoding HMG-CoA reductase). Negative selection was facilitated by the *hpt* gene encoding hypoxanthine phosphoribosyltransferase, rendering stains susceptible to the base analog 8-azahypoxanthine. We also demonstrated genetic complementation using an integration vector. These tools should be generally useful in related organisms.

We addressed two novel features of the *nif* gene cluster of FS406. First, we tested the possible roles of the three novel open reading frames *hp1*, *hp2*, *hp3*, and *fd* in nitrogen fixation. In addition to the presence of these genes in FS406, each gene is present in at least one additional species of hyperthermophilic methanogen (Fig. 1). *hp1* and *hp2* are also present in the moderate thermophile Methanothermococcus okinawensis, and *hp1* is present in Methanococcus aeolicus (*M. aeolicus*, though mesophilic, branches separately from other mesophilic *Methanococcus* species and is related to *M. okinawensis*, [19].) The persistence of these genes, especially in thermophilic members of the Methanococcales that fix nitrogen, suggests that they do confer an adaptive advantage. However, our results could not support the hypothesis that the genes play any role in nitrogen fixation, at least under the conditions we used for growth. The genes may instead play a role unrelated to nitrogen fixation, despite their presence in a cluster of *nif* genes. Alternatively, they could still play a role in nitrogen fixation under some conditions in nature. For example, the *hp1*, *hp2*, and *hp3* gene products could perform chaperone-like functions that are not apparent under the temperatures and conditions we used for diazotrophic growth in the lab. Deletion of the gene encoding an apparent ferredoxin also had no effect. A source of low-potential electrons is required for nitrogen fixation, and the ferredoxin encoded in the *nif* cluster could still serve this function if another ferredoxin can functionally replace it. Ferredoxins are abundant in genomes of methanogens and some may be able to function promiscuously. A ferredoxin-encoding gene is absent from the *nif* gene clusters of other diazotrophic methanogens as well, including the well-characterized *M. maripaludis* (8).

The second novel feature of the *nif* gene cluster of FS406 relates to the biosynthesis of the nitrogenase cofactor, which requires *nifB*, *nifE*, and *nifN* in well-characterized diazotrophs (7). NifB carries out the first step specific to FeMoco synthesis, the generation of the 8Fe core precursor. NifB is not encoded in the *nif* cluster of FS406, but is present elsewhere in the genome (MFS40622_0164). Biochemical studies have shown that NifB from species related to FS406 does indeed function as expected in cofactor synthesis (20, 21), and it is likely that the homolog in FS406 does as well. NifB in FS406 is encoded in a cluster of other genes with no apparent specific relation to nitrogen fixation, and a conserved operator sequence known to function in repression of nitrogen fixation and assimilation genes in methanogens is absent (12, 13). This suggests that *nifB* may not be derepressed solely under nitrogen fixing conditions and nitrogen fixation may not be its sole function (22). Indeed, close homologs are found in nonnitrogen fixers, including *M. jannaschii*.

A more surprising observation is the absence of *nifN*. In well-characterized diazotrophs, NifE and NifN are both essential for the formation of the final FeMoco and its delivery to NifDK (7). However, phylogenetic analyses have implicated ancestral nitrogenases or proto-nitrogenases that may have existed in ancient organisms with only *nifHDKB* or only *nifHDKBE* (4–6). *nifE* and *nifN* evidently originated in a single event by gene duplication from *nifD* and *nifK*, or first *nifE* originated from *nifD* followed by the origin of *nifN* from *nifK*. *nifHDKB* alone are found in certain representatives of the Chloroflexi, although nitrogen fixation in these organisms remains unconfirmed, whereas *nifHDKBE* alone are found in certain thermophilic Firmicutes as well as FS406 and its relatives, and diazotrophy is confirmed (6, 23). In FS406, as suggested for an ancestral *nifHDKB* organism, NifD and NifK could carry out the synthesis of the nitrogenase cofactor as well as catalyzing nitrogen fixation. If this is the case, NifE may not be necessary. However, our results, suggesting that NifE could play a role, may indicate that NifE alone is responsible for the final step in the cofactor synthesis. In either case, it would be of great interest to determine if the structure of the cofactor is the same as FeMoco or is instead some form of a proto-Mo-cofactor as hypothesized (6). The question also remains, what is the function of Hp4, which is encoded adjacent to *nifE* in an apparent operon. Hp4 could take the place of NifN, a NifE-Hp4 complex carrying out the function that NifEN plays in typical diazotrophs. However, Hp4 has no discernible homology with NifN, nor was it absolutely required in our growth studies. As a final possibility, NifE could combine with NifK (homologous with NifN) to carry out FeMoco biosynthesis. In this case, Hp4 could somehow facilitate the interaction of NifE with NifK. Biochemical studies are needed to sort out these possibilities. In any case, FS406 appears to illustrate the smallest set of nitrogen fixation-specific genes yet demonstrated in a confirmed diazotroph. *nifH*, *nifD*, *nifK*, and *nifE* seem clearly dedicated to nitrogen fixation, while the *nifB* homolog may have an additional function that is currently unknown. It has been proposed (4–6) that biological nitrogen fixation first arose in an ancestor of hydrogenotrophic methanogens, of which *Methanocaldococcus* and related genera are members. FS406, with its minimal gene set, may represent an early stage in the evolution of nitrogen fixation.

## MATERIALS AND METHODS

### Growth and media.

FS406 and its derivatives were grown essentially as described for *M. maripaludis* (16) with the following modifications. McCas medium was modified by removing tungsten and Casamino Acids, adding PIPES HCl to a final concentration of 50 mM, and adjusting the pH to 6.0 with NaOH at room temperature. For routine growth and construction of strains, NH_4_Cl (10 mM) was included and the gas atmosphere was 80% H_2_ and 20% CO_2_ at a pressure of 40 psig (275 kPa). For diazotrophic growth, NH_4_Cl was omitted; tubes were prepared initially with a gas atmosphere of 80% N_2_ and 20% CO_2_ at 30 psig (207 kPa), depressurized to 0 psig before inoculation, and finally pressurized to 40 psig with 80% H_2_ and 20% CO_2_. Liquid cultures were incubated in Balch tubes (24) placed horizontally on their sides with reciprocal shaking at 50 rpm in a water bath at the temperatures indicated. For plates, Noble Agar (2% wt/vol) was used. 8-aza-hypoxanthine (250 μg/mL) was added for negative selection and neomycin sulfate (300 μg/mL) for positive selection as needed. In some cases, for positive selection simvastatin was used instead of neomycin. An anaerobic stock solution of 5 mM simvastatin in 50% (vol/vol) ethanol was made in the anaerobic glove box and filter sterilized through 0.20 μm autoclaved nylon filter discs (Cole Palmer Catalog number 02915-16) into sterile serum bottles. After genetic transformation, initial selection was done at a final concentration of 20 μM simvastatin. For maintenance of selected plasmids in strains, 10 μM simvastatin was used. Genetic transformations (16, 17) and incubation of agar plates were conducted at 70°C in sealed steel incubation vessels (Fig. S4) containing an atmosphere of 80% H_2_ and 20% CO_2_ at 20 psig. Optical density was measured at 660 nm in a Spectronic 20.

### Construction of pJAL2 (Pmj*rpo*::*kat* Pmj*mtr*::*hpt*).

PCR primers are listed in Table S1. To generate the *M. jannaschii rpo* promoter fused to the *kat* gene (P*rpo*::*kat*), PCR products containing the *M. jannaschii rpoH* promoter (MJ1039) and a thermostable *kat* gene (encoding kanamycin nucleotidyltransferase) from plasmid pMK18 ([25], purchased from Biotools) were digested with NdeI and ligated. The product was PCR amplified, digested with SpeI and XbaI, and cloned into a holding vector. Similarly, to generate a P*mtr*::*hpt* fusion, PCR products containing the *M. jannaschii mtrE* (MJ0847) promoter and the FS406 *hpt* (MFS40622_0597) gene were digested with NsiI, ligated, amplified, digested with SpeI and XbaI, and cloned into a holding vector. The two promoter::gene fusions were PCR amplified with the same primers with the exception that the XbaI and SpeI restriction sites of the reverse primer for the *kat* and the forward primer for the P*mtrE* were replaced with HpaI restriction sites. These gene fusions were then digested with HpaI, and ligated, producing a *kat*-*hpt* fragment joined at the HpaI site. PCR products containing E. coli Ori and *amp* regions were digested with PvuII and ligated. The resulting *ampR* - Ori region, and the *kat-hpt* fragment were amplified, digested with EcoRV and HindIII, and ligated to create pJAL2.

### Construction of pJALv3s1 (Pmj*mtr*::*kat* Pmj*mcr*::*hpt*).

To create the *M. jannaschii mtrE* promoter fused to *kat* (from pMK18), the PCR products containing the *mtrE* promoter and *kat* genes were cut with NsiI and PstI, respectively, and ligated. To fuse the *M. jannaschii mcrB* promoter (MJ0842) to the FS406 *hpt* gene, PCR products were digested with NsiI and ligated. Both promoter fusions were amplified, digested with PvuII, and ligated to create a *kat*-*hpt* fragment fused to their respective promoters. This fragment was amplified and digested with NotI and HindIII. The Ori-*ampR* region was obtained from pJAL2 with NotI, HindIII and EcoRI HF. The largest band (containing NotI-HindIII ends) was gel purified and ligated with the *kat*-*hpt* fragment to produce pJALv3s1.

### Construction of pLKH (Pmj*mtr*::*cohtk* Pmj*mcr*::*hpt*).

A DNA fragment containing Pmj*mtrE* fused to *cohtk* was synthesized (Genewiz, Inc.). *cohtk* is a codon optimized (for FS406) gene encoding a highly thermostable kanamycin nucleotidyltransferase generated by directed evolution resulting in 19 amino acid replacements and having full activity at 75°C and partial activity up to 80°C (18). A DNA fragment containing Pmj*mcrB* fused to FS406 *hpt* was also synthesized (Genewiz, Inc.). The two DNA fragments were digested with NotI and HindIII and ligated to HindIII and NotI-digested Amp-Ori region from pJALv3S1 to produce pLKH.

### Construction of the integration vector pLHI2 (Pmj*mcr*::*hpt* Pmj*mtr*::*hmgA*).

The *M. jannaschii hmgA* (MJ0705) gene encoding HMG-CoA reductase was used to confer simvastatin resistance. PCR-amplified *M. jannaschii hmgA* and a PCR-amplified region of pLKH without the *cohtk* gene were ligated at overlapping ends using an *in vivo* DNA assembly method (26) resulting in pLHH. To serve as a neutral site for integration, a PCR product containing the last 500 bp of the FS406 *nrpR* gene (MFS40622_0114) was digested with NotI and XbaI and cloned into pLHH to give pLHI2.

### Construction of complementing plasmid pLIH2*hp4*.

The *hp4* gene (MFS40622_0040) was PCR amplified with its native promoter, digested with AscI, and cloned into AscI-digested pLIH2. Primers TLP535 and TLP440 were used to screen for the Php4::*hp4* insert, then primers TLP 533 and TLP 440 were used to test for directionality.

### Construction of strain ML100 (Δ*hpt*).

Flanking regions (0.5 kb) of the FS406 *hpt* gene (FS40622_0597) were PCR amplified from genomic DNA using primer sets 99/100 and 101/102. Digestion with AscI and ligation resulted in an in-frame deletion containing the AscI site between the *hpt* start and stop codons. The ligated product was amplified and the product was gel-purified and used for transforming FS406. Colonies were selected with 8-aza-hypoxanthine and screened for a shortened *hpt* gene.

### Construction of strain ML111 (Δ*nifD*).

DNA fragments (0.5 kb) upstream and downstream of *nifD* (MFS40622_0034) were obtained by PCR, digested with AscI, and ligated. The ligated fragment was amplified, digested with XbaI and HindIII, and cloned into XbaI and HindIII digested pJALv3S1 resulting in pJALΔ*nifD.* The deletion was in frame with 48 codons remaining between the start and stop codons. The *cohtk*-*hpt* fragment from pLKH was PCR amplified, digested with AscI, cloned into pJALΔ*nifD*, and screened for directionality by restriction digests (*cohtk*-*hpt* in the same direction as the *nifD*) producing pJALΔ*nifDcohtkhpt.* This plasmid was then linearized with XbaI and transformed into ML100. The transformation mixture was plated with selection for neomycin resistance. Colonies were picked and screened by PCR for a double recombinant with a truncated *nifD* containing the *cohtk*-*hpt* cassette. A second transformation was done with linearized pJALΔ*nifD* and selected on plates with 8-aza-hypoxanthine to generate strain ML111 containing a markerless in frame deletion of *nifD*.

### Construction of strain ML200 (Δ*hp1*-*hp2*-*hp3*-*fd*::*cohtk*-*hpt*).

DNA fragments upstream of *hp1* and downstream of *fd* were obtained by PCR, digested with AscI, and ligated, resulting in a fragment containing the first 19 codons of *hp1*, a TAA stop codon, an AscI site, a glycine codon, and finally the last 7 codons of the *fd* gene (Fig. S5). The ligated fragment was amplified, digested with XbaI and HindIII, and cloned into pJALΔ*nifD* (with the XbaI – Δ*nifD* – HindIII portion digested out) via the XbaI HindIII sites resulting in plasmid pL delta hp1ferro. The *cohtk*-*hpt* cassette was amplified from pLKH using primers TLP521 and TLP522 resulting in a product with AscI sites on both ends. This fragment was cloned into plasmid pL delta hp1ferro at the AscI site. Distinct large and small colony sizes of E. coli transformants were obtained. DNA from a large colony transformant was linearized with XbaI, transformed into ML100, and plated with neomycin selection, resulting in strain ML200 with the region from *hp1* to *fd* replaced with *cohtk*-*hpt*.

### Construction of strain ML103 (Δ*hp4*Δrbs*nifE*).

Left and right flanking regions of *hp4* were PCR amplified (primer sets TLP377/TLP378 and TLP379/TLP380, respectively), digested with AscI, and ligated, resulting in an in-frame deletion of the *hp4* gene containing the start and stop codons with the AscI sequence (plus one extra nucleotide to make the deletion in-frame) in between. The deletion also removed the ribosome binding site and start codon of *nifE*. This construct was cloned into the XbaI NotI site in pJAL2 and the resulting plasmid was transformed into ML100 selecting for neomycin resistance. Outgrowth was allowed to occur in nonselective medium, and the mixture was then plated on medium containing 8-azahypoxanthine (16). Primers TLP 377 and TLP 380 were then used to screen candidate colonies for the deletion (Fig. 3B).

### Construction of strain ML104 (Δ*hp4*).

Primer pairs TLP377/TLP378 and TLP449/TLP380 were used to amplify the upstream and downstream regions of *hp4,* respectively. The fragments were digested with AscI, gel purified, ligated, and amplified with the TLP377/380 primer pair, resulting in a construct with an in-frame deletion of *hp4* with a start codon, a stop codon, and an in-frame intervening sequence with an AscI site. This construct was digested with NotI and XbaI and directionally cloned into pJAL2 digested with the same enzymes to give pJAL2 Δ*hp4*. This plasmid was transformed into ML100 selecting for neomycin resistance. Outgrowth was allowed to occur in nonselective medium, and the mixture was then plated on medium containing 8-azahypoxanthine (16) resulting in ML104 containing an in-frame deletion of *hp4* while leaving the rbs and the *nifE* gene intact (Fig. 3C).

### Complementation of Δ*hp4*.

Plasmids pLHI2 and pLIH2*hp4*were transformed into strain ML104 (Δ*hp4*) and selected with simvastatin.

Genome sequences were downloaded from the NCBI site (https://ftp.ncbi.nlm.nih.gov/genomes/) and viewed with Artemis (https://www.sanger.ac.uk/tool/artemis/).
