# Supplementary material for: A Genetic Study of Nif-Associated Genes in a Hyperthermophilic Methanogen
Source: Microbiol Spectr. 2022 Feb 2;10(1):e02093-21. doi: 10.1128/spectrum.02093-21 (PMC8809328; doi:10.1128/spectrum.02093-21)

Supplemental Material

Supplemental figures

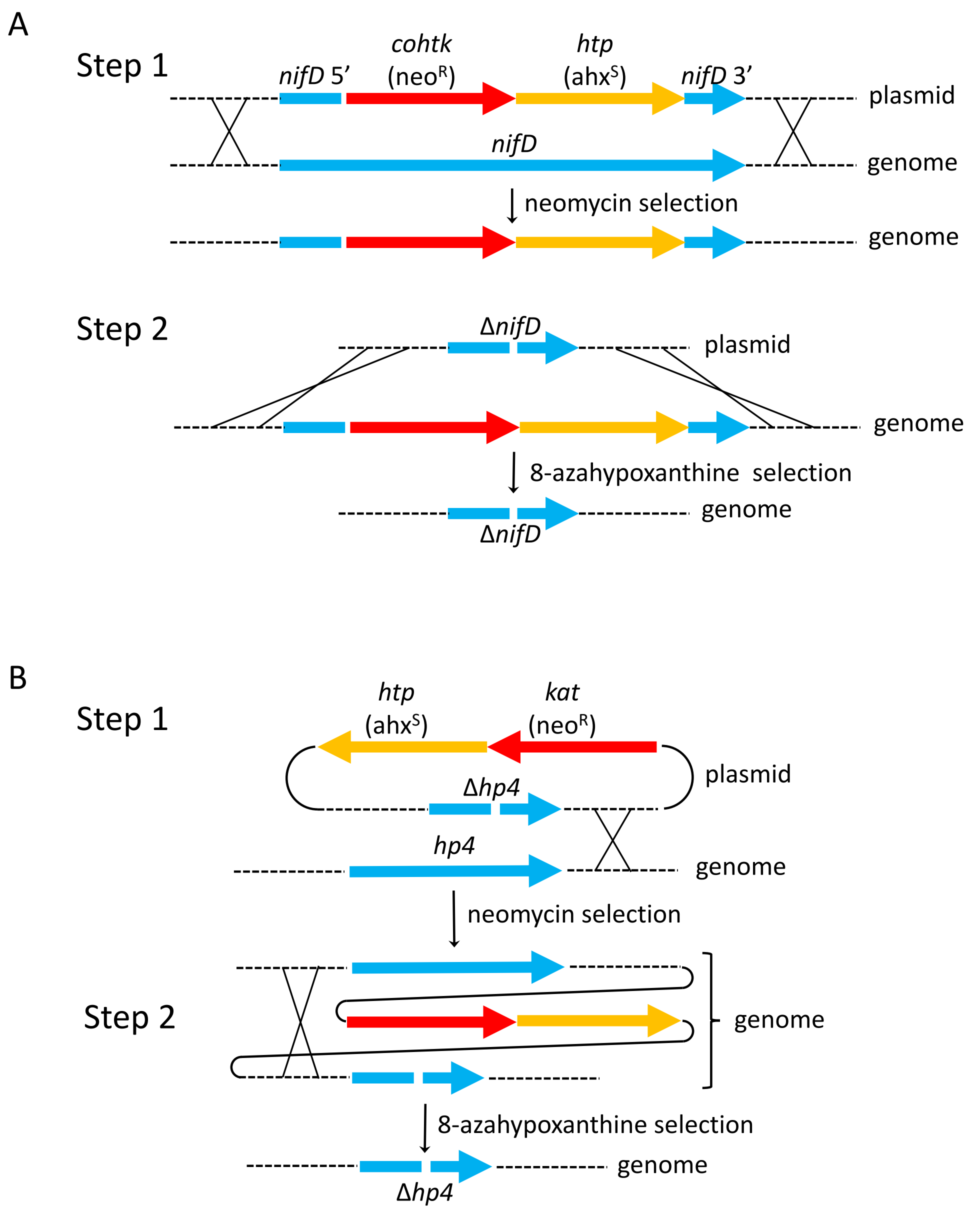

Fig. S1. Construction of Δ*nifD* (A) and Δ*hp4* (B) mutants. Dashed lines represent flanking sequences. Solid lines represent vector sequences. "X" shapes represent homologous recombination. ahx^S, 8-azahypoxanthine-sensitive. In (A), two transformations are done, and two double homologous recombinations occur. In (B), one transformation is done, and a homologous recombination event occurs resulting in a merodiploid. In step 2 a second recombination event occurs. Each step depicts one of two possible recombination events. The procedure yields either the mutant allele as shown, or the wild type allele, depending on which recombination events occur. The mutant allele is identified by screening.

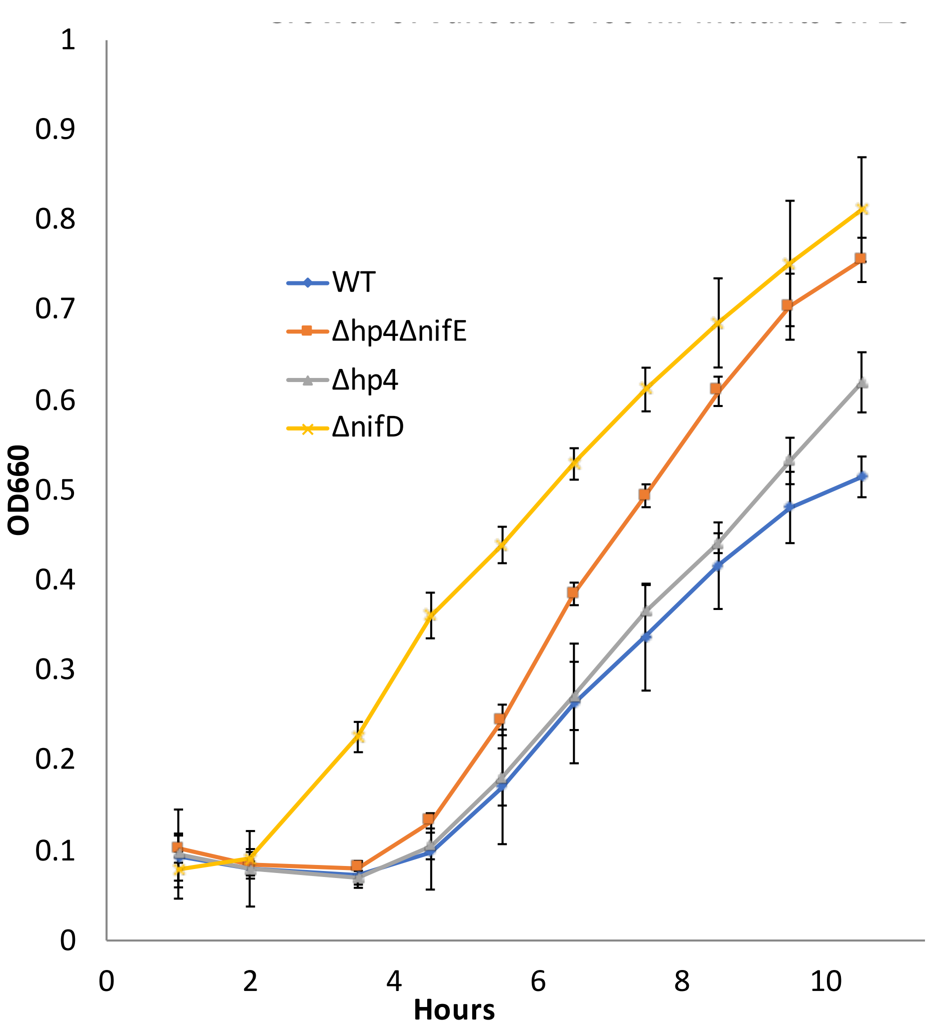

Fig. S2. Growth of various FS406 *nif* mutants on NH_4^+ at 85°C. Averages of three cultures and standard deviations are shown.

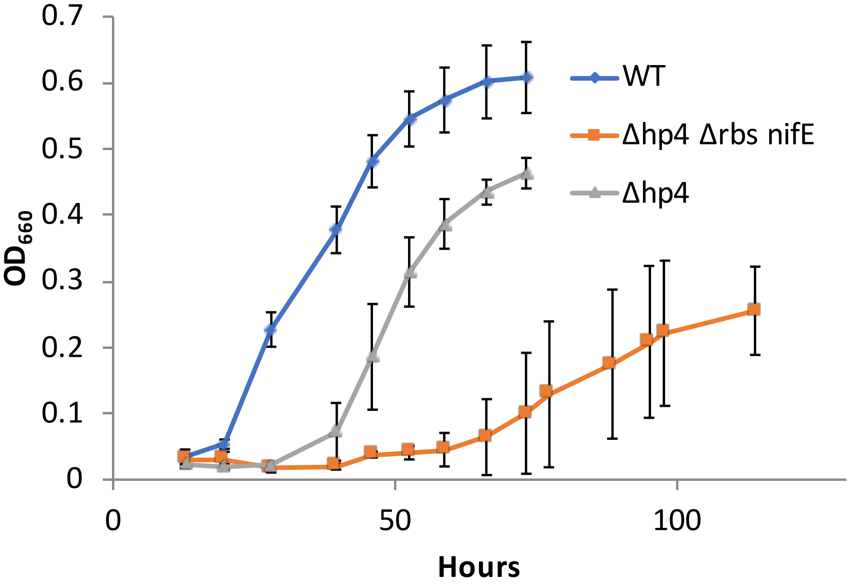

Fig. S3. Diazotrophic growth of Δ*hp4*Δrbs*nifE* and Δ*hp4* mutants inoculated with diazotrophically grown cultures. Growth temperature was 75°C. Averages of three cultures and standard deviations are shown.

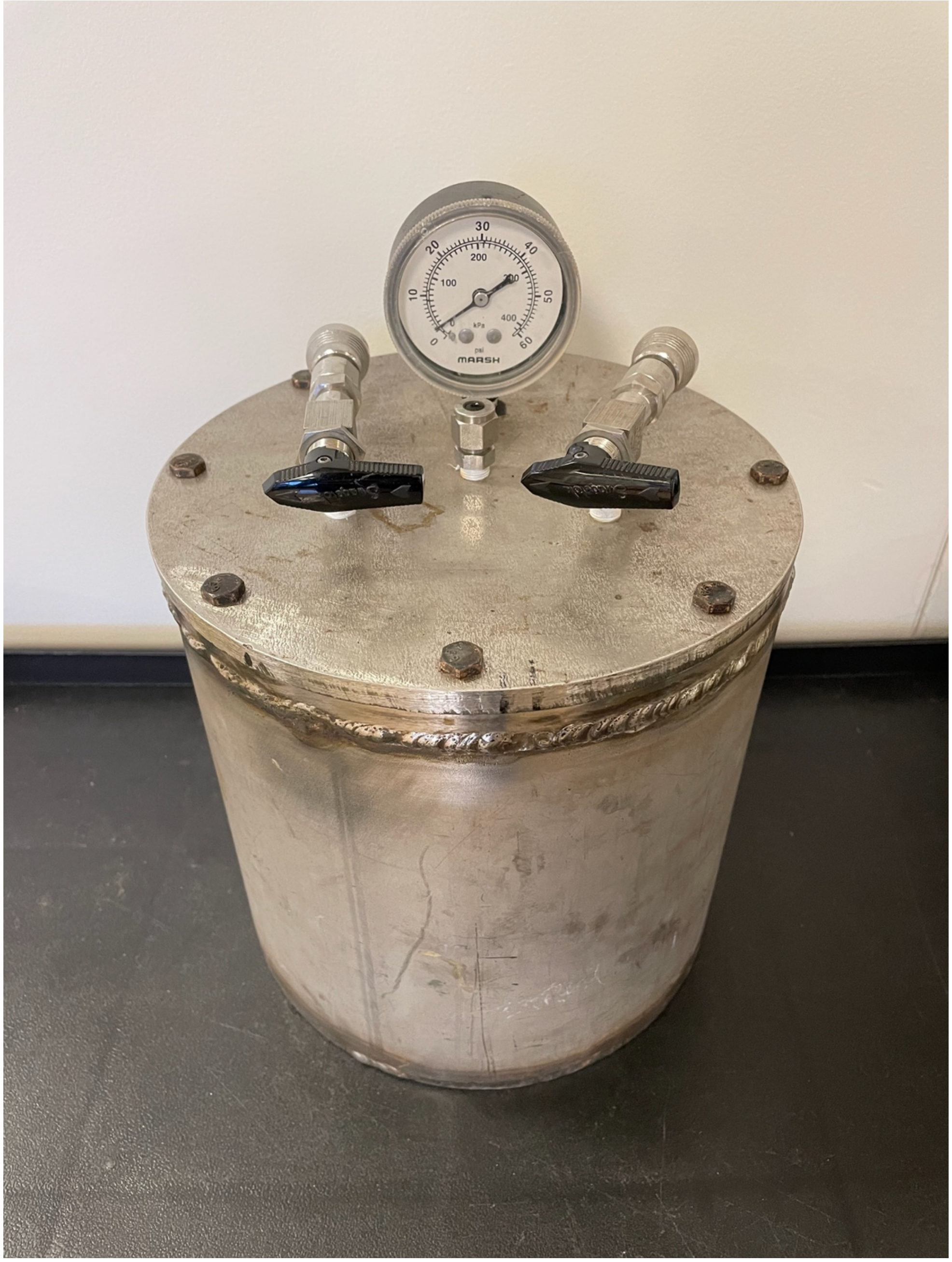

Fig. S4. Steel incubation vessel for anaerobic incubation of agar plates

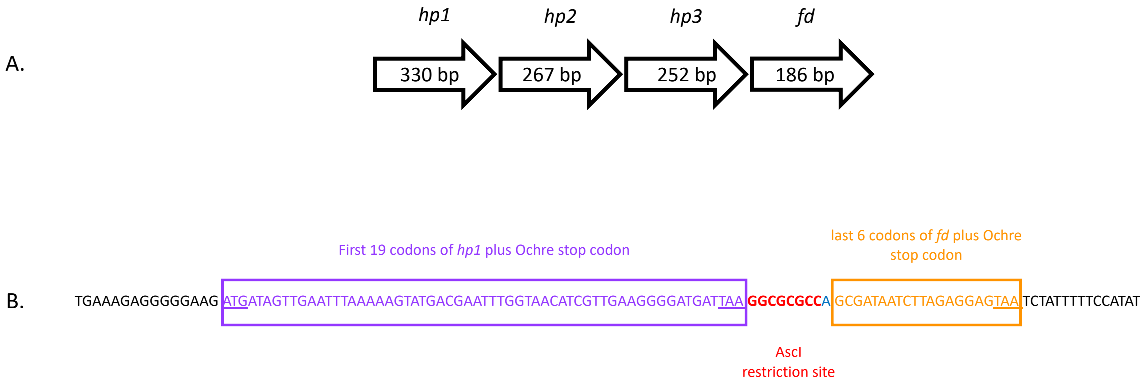

Fig. S5. (A) Wildtype *hp1* to *fd* gene cluster of FS406. Numbers within arrows (*hp1*, *hp2*, *hp3, fd*) represent total number of nucleotides. (B) Δ*hp1* to *fd* region of plasmid pL Δhp1ferro . Letters in black are the upstream sequences of *hp1* and downstream sequences of *fd* respectively. Letters in purple represent in-frame deletion of *hp1* with 18 codons between the start and stop codons. Red letters represent the AscI restriction site. Blue letter represents nucleotide added during cloning to allow the AscI site (plus the A nucleotide) to code for the amino acids, Gly, Ala, and Pro. Orange letters represent the last 7 codons of the *fd* gene. Start and Stop codons are underlined.

Table S1. PCR primers

Primer name	Sequence	Product
TLP318	AAA CTA GTA AAT AAA GAA ATA TTT TTA TAG GAC (SpeI)	*M. jannaschii rpoH*
TLP368	ATT TTT CAT ATG TAC TCC TCC CTA CCA TTT TG (NdeI)	promoter (MJ1039)
TLP327	GTG AGG CAT ATG AAC GGA CCA ATA ATA ATG ACT AGA G (NdeI)	*kat* gene from pMK18
TLP321	AAT CTA GAT CAA AAT GGT ATG CGT TTT GAC (XbaI)	
TLP 314	AAA CTA GTA CAA AAA CTT AAA TTC TGA C (SpeI)	*M. jannaschii mtrE*
TLP 315	AAA TGC ATT CAC CTC ATT TTA CAT AAG AC (NsiI)	promoter (MJ0847)
TLP316	AAA TGC ATG TTT ATA AAG TGC TAT AGT G (NsiI)	FS406 *hpt* gene
TLP367	AAT CTA GAT TAA TCC CTT AAA ATA ACA AC (XbaI)	(MFS40622_0597)
TLP386	CGG TAT GAT ATC AAA TAA AGA AATY ATT TTT ATA GGA C (EcoRV)	*M.jannaschii rpoH* promoter (MJ1039)
TLP 373	TCG TGC CAG CTG CAT TAA TGA ATC GGC C (PvuII)	Ori region from
TLP 374	TCT GCA GAT ATC CAT CAC ACT GGC GGC C (EcoRV)	pcruptneo III
TLP 375	TAA AAG CTT GGG GAA ATG TGC GCG GAA CC (HindIII)	Amp region from
TLP 376	TTA ATG CAG CTG TTA ATC AGT GAG GCA CCT ATC TC (PvuII)	pJAR50CT
TLP 456	ATA TAT GCG GCC GCG ATG GAT ATC ACA AAA ACT TAA ATT CTG ACA TTG (NotI)	*M. jannaschii mtrE* promoter
TLP 453	ATA TAT ATG CAT CAC CTC ATT TTA CAT AAG AC (NsiI)	
TLP 362	AAC TGC AGA TGA ATG GAC CAA TAA TAA TGA CTA G (PstI)	*kat* gene
TLP 455	ATA TAT CGA TGC TCA AAA TGG TAT GCG TTT TGA C (PvuI)	
TLP 451:	ATA TAT GTT AAC CTG TTA TTT TTA ATA AAA AAT TTC AGA TAG (HpaI)	*M. jannaschii mcrB*
TLP 452	ATA TAT ATG CAT ACA TAT TGG TTC GAT ATT AGT TAT CGC (Nsi)	promoter (MJ0842)
TLP 316	AAA TGC ATG TTT ATA AAG TGC TAT AGT G (NsiI)	FS406 *hpt* gene
TLP 387I	TCG TGC AAG CTT TTA ATC CCT TAA AAT AAC AAC (HindIII)	
TLP 520	ATA TAT GCG GCC GCG ATG GAT ATC ATT ATG TTG ATA TTG (NotI)	Pmtr:cohtk –
TLP 387I	TCG TGC AAG CTT TTA ATC CCT TAA AAT AAC AAC (HindIII)	Pmcr:hpt
TLP 518I	ATA TAT AAG CTT GGG GAA ATG TGC GCG GAA CC (HindIII)	Amp-Ori from
TLP 519I	GAT ATC CAT CGC GGC CGC TCG AGC ATG CAT CTA GAG C (EcoRV NotI rev)	pJALv3S1
TLP 527	CAA GGA TGT CAT TAT AAT TTT CCA TCC TAT CAC CTC ATT TTA CAT AAG AC	pLHI from pLKH
TLP 528	GCT CAT CAA GAA CTC GGT AGA TAA CTG TTA TTT TTA ATA AAA AAT TTC AGA TAG	

TLP 525	GTC TTA TGT AAA ATG AGG TGA TAG GAT GGA AAA TTA TAA TGA CAT CCT TG	*M. jannaschii hmgA* gene
TLP 526	CTA TCT GAA ATT TTT TAT TAA AAA TAA CAG TTA TCT ACC GAG TTC TTG ATG AGC	
TLP 533	ATA TAT GCG GCC GCT CTG ACA GAT TTG GAA TTG TTG AGG AAG AAG (NotI)	Last 500bp of FS406 *nrpR* region
TLP 534	ATA TAT TCT AGA AAG GCG CGC CCA CAA TCC ATA GAG GTT TCA CC (XbaI)	
TLP 535	ATATATGGCGCGCCATGTTTTAAACTATAAGGAAATAAGTTTCC (AscI)	native FS406 *hp4* and promoter region
TLP 440	ATA TAT GGC GCG CCT CAT ACT ATC TCC TTC AAT ACC TC (AscI)	
Primer 99	AAA AGC GGC CGC CAG AGT TAG AAT CTT AGG GAA GG (NotI)	FS406 Δ*hpt*
Primer 100	AAA AGG CGC GCC ACA ATC TTC CAC CTC CAC TAT AGC AC (AscI)	
Primer 101	AAA AGG CGC GCC TAA ATA TGG AAA AGC CCT TAA AAT C (AscI)	
Primer 102	AAA ATC TAG AAT GAT TTT TGT TGG ATT TAT CTC TC (XbaI)	
TLP 478	ATA TAT TCT AGA GGA AAC TTT GGA GAT GGG (XbaI)	FS406 Δ*nifD*
TLP 479	ATA TAT GGC GCG CCT TAA GCC GGG ATT ATC TC (AscI)	
TLP 480	ATA TAT GCG GCC GCG AAT TTA AGA AAA AAG ATA TTC C (NotI)	
TLP 503	ATA TAT AAG CTT GGA ATT ATT GGG AGT TGA GCT TTC (HindIII)	
TLP 484	ATA TAT TCT AGA GTT GGA ATC TTT GGG GAT CC (XbaI)	upstream of hp1
TLP 485	ATA TAT GGC GCG CCT TAA TCA TCC CCT TCA AC (AscI)	
TLP445-2	ATA TAT GGC GCG CCA GCG ATA ATC TTA GAG G (Asci)	downstream of ferredoxin
TLP446-2	TAT ATA AAG CTT CTT TGA CCT TAA CTT C (HindIII)	
TLP 521	ATA TAT GGC GCG CCA TAT CAT TAT GTT GAT ATT GTT AAG C (AscI)	*cohtk-hpt* cassette from pLKH
TLP 522	ATA TAT GGC GCG CCT AAA CAA ATA GGG GTT CCG CGC (AscI)	
TLP377	AAT CTA GAC GAG GAG TTA GTT GAG GAA G (XbaI)	Δ*hp4*Δrbs*nifE* ML103
TLP378	AAG GCG CGC CCA TGC TTT CAA CCT CCT GTG TCC (AscI)	
TLP379	AAG GCG CGC CCT GAA TGA AAT AAA AGT CCT TCC (AscI)	
TLP380	AAG CGG CCG CTT AGT CTA AAT AAT GCC TTC ATT GC (NotI)	
TLP377	AAT CTA GAC GAG GAG TTA GTT GAG GAA G (XbaI)	Δ*hp4* ML104
TLP378	AAG GCG CGC CCA TGC TTT CAA CCT CCT GTG TCC (AscI)	
TLP449	ATA TAT GGC GCG CCC TGA GGT ATT GAA GGA GAT AGT ATG AAT G (AscI)	
TLP380	AAG CGG CCG CTT AGT CTA AAT AAT GCC TTC ATT GC (NotI)	
TLP 482	ATA TAT GGC GCG CCA CAA AAA CTT AAA TTC TG (AscI)	Pmjmtr::*kat* - Pmjmcr::*hpt* from pJAL v3S1
TLP 490	ATA TCT GCG GCC GCT TAA TCC CTT AAA ATA ACA ACT TTC C (NotI)	

Strain list

Strain	Description
ML100	Δhpt
ML103	Δhpt Δhp4 rbs nifE
ML104	Δhpt Δhp4
ML111	ML100 ΔnifD
ML112	ML104 and pLIH2
ML113	ML104 and pLIH2hp4
ML114	ML103 and pLIH2
ML115	ML100 and pLIH2
ML200	ML100 Δhp1-ferredoxin

Plasmid diagrams and descriptions

pJAL2 contains a thermostable kanamycin nucleotidase, *kat*, expressed from the *M. jannaschii rpoH* promoter and the FS406 *hpt* gene driven by the *M. jannaschii mtrE* promoter.

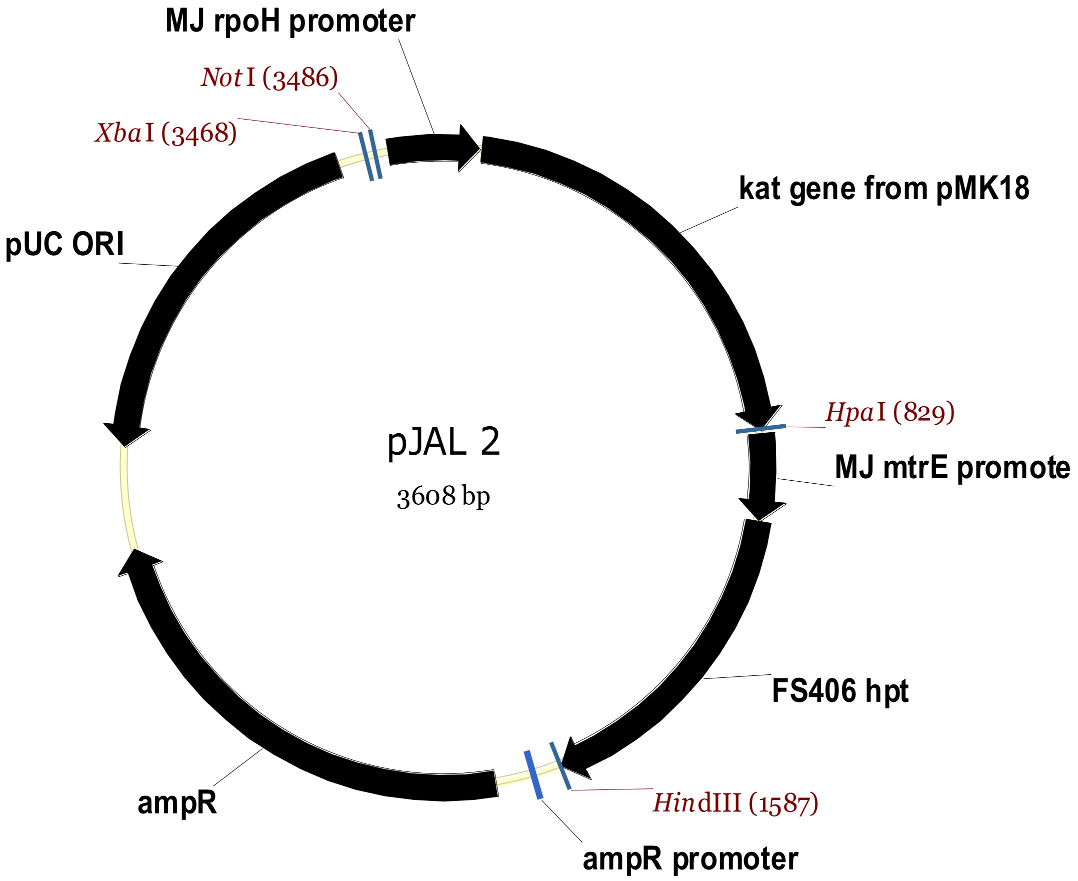

pJALV3S1 also contains the thermostable *kat* and FS406 *hpt* genes but they are driven by the *M. jannaschjii mtrE* and *mcrB* promoters respectively

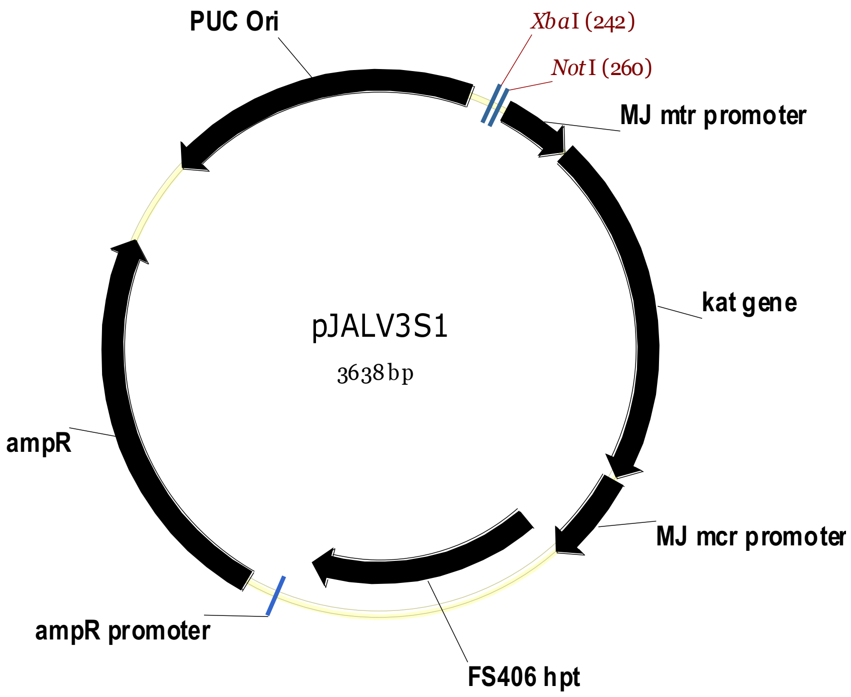

pLKH contains the high temperature-evolved *kat* gene *cohtk* driven by the *M. jannaschii mtrE* promoter and the FS406 *hpt* gene driven by the *M. jannaschii mcrB* promoter.

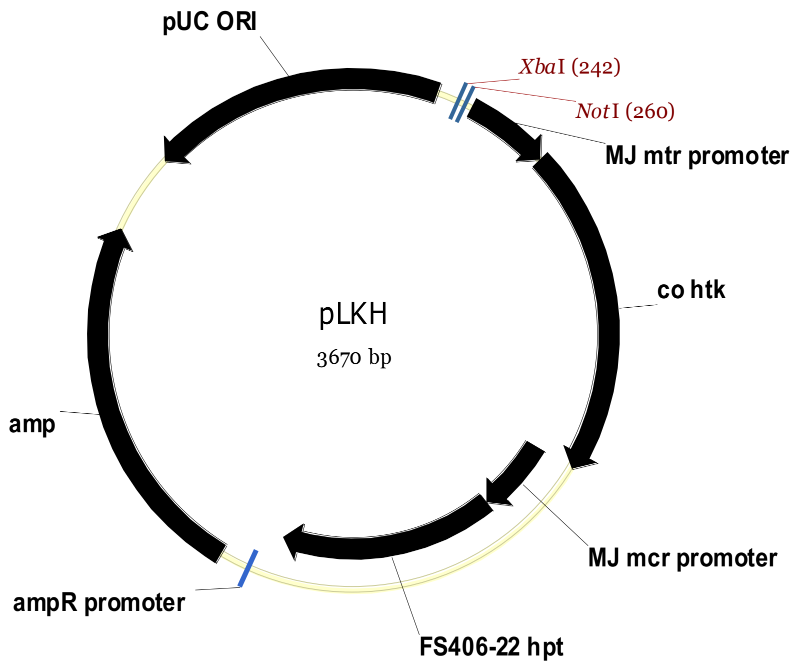

pLHH contains the *M.jannaschii* HMG CoA reductase gene driven by the strong *M.jannaschii mtrE* promoter

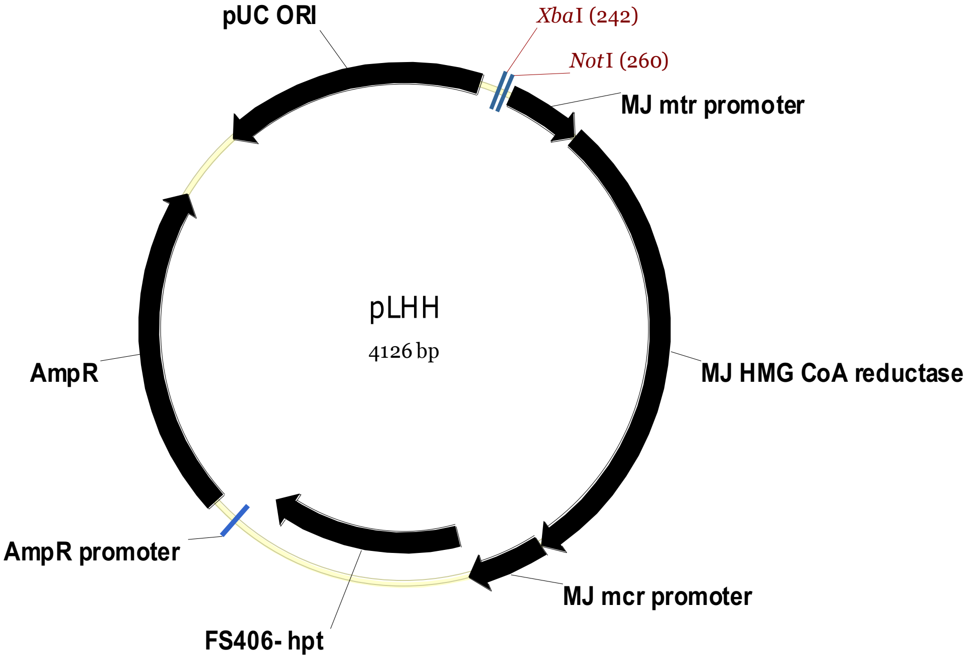

pLIH2 is an integration vector with the *M. jannaschii* HMG CoA reductase gene used for simvastatin selection. It also has about 500 bp of the intergenic region downstream of the FS406 *nrpR* gene for homologous recombination.

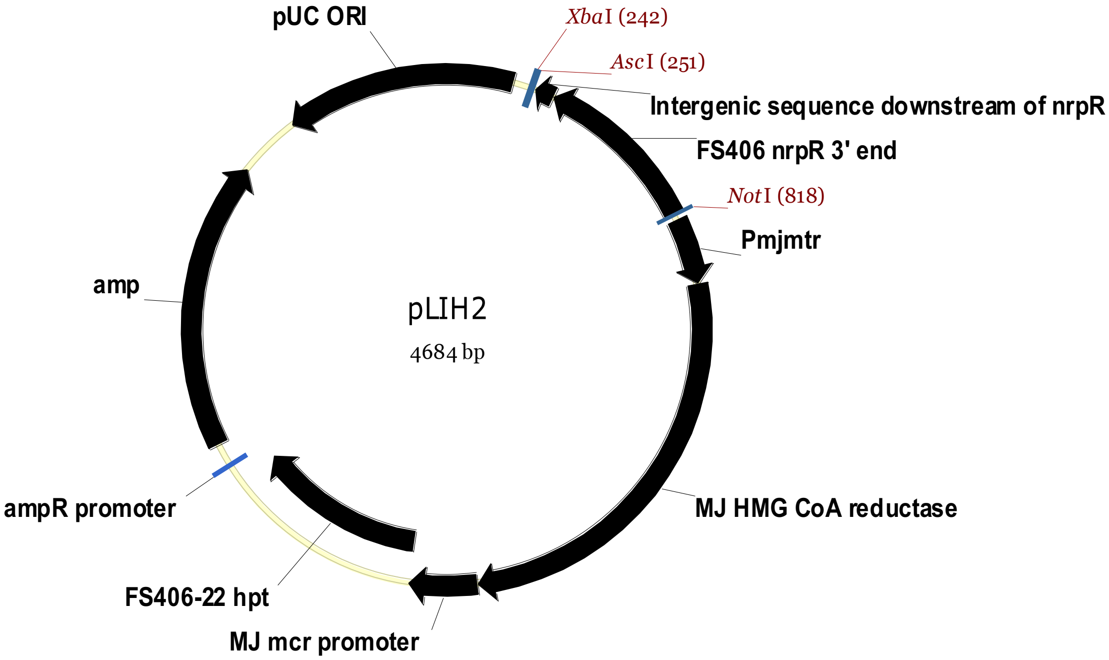

pLIH2hp4 is pLIH2 (above) with the FS406 *hp4* gene including its upstream promoter region.

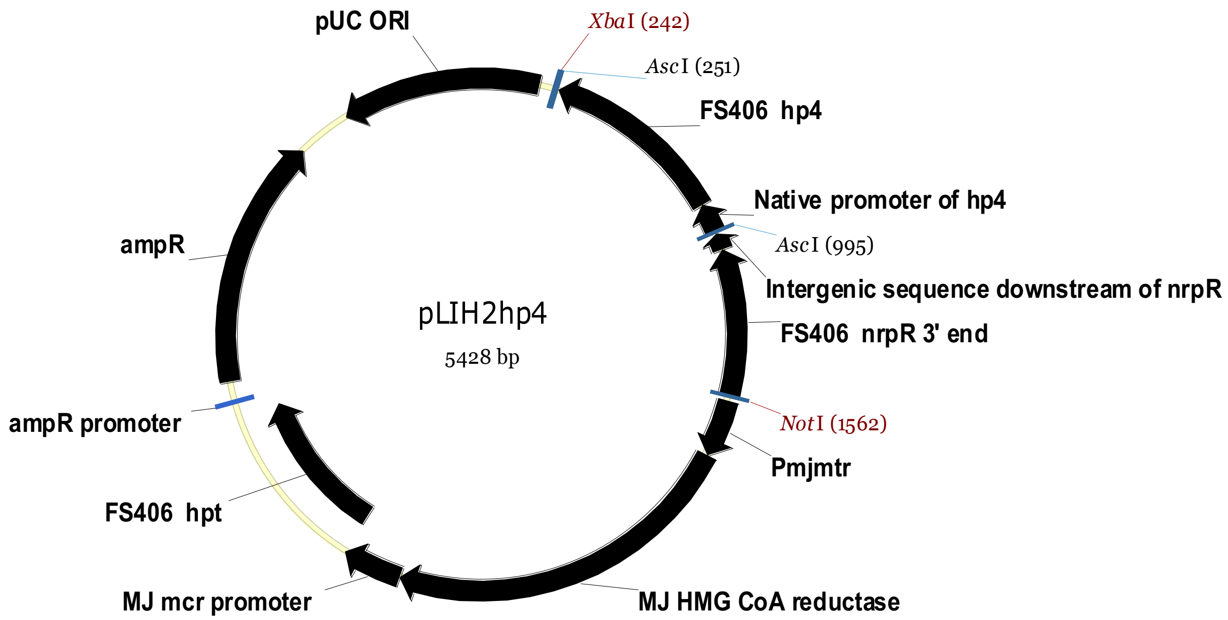

pJAL del nifD contains an in-frame deletion of FS406 *nifD*

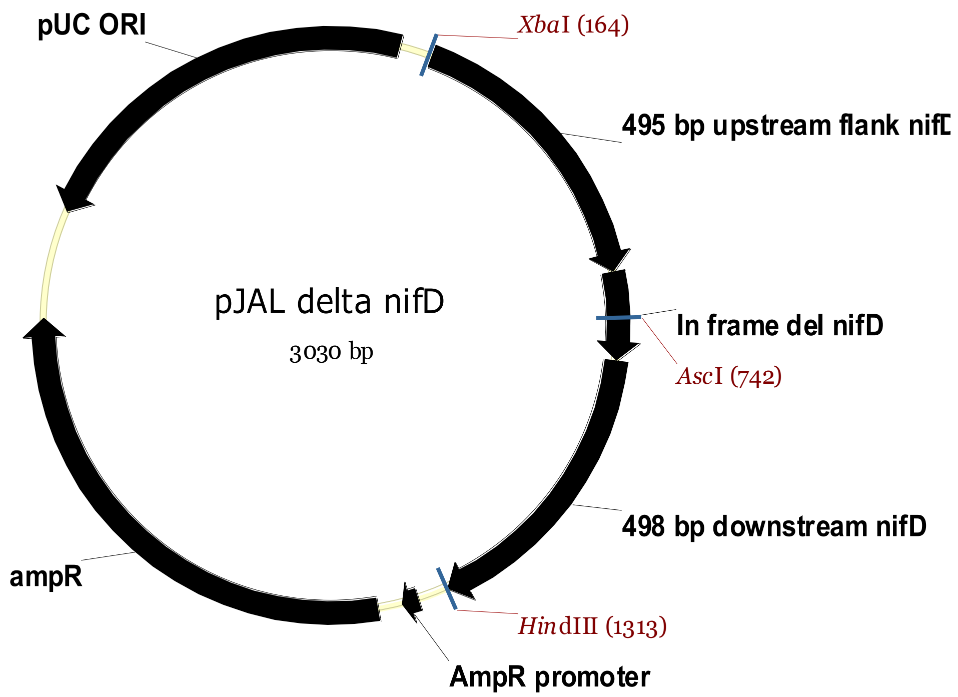

pL delta nifD cohtkhpt contains the high temperature-evolved *kat* gene *cohtk* and the FS406 *hpt* gene cassette inserted into the in-frame deletion of *nifD* at the AscI site.

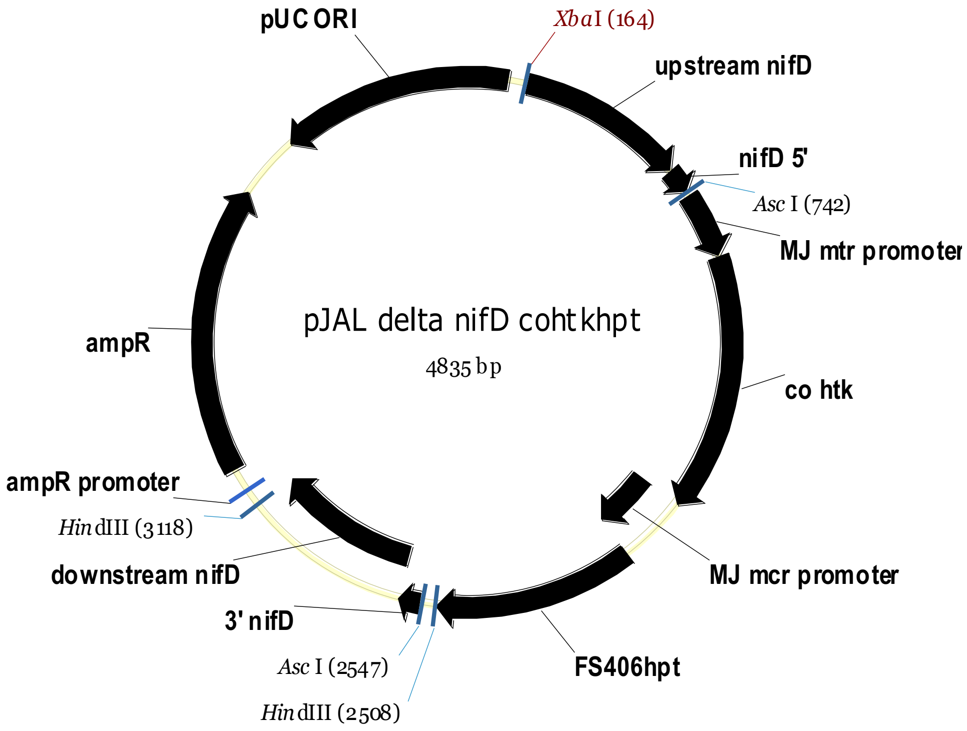

Supplement: SUPPLEMENTAL FILE 1 — Supplemental material. Download SPECTRUM02093-21_Supp_1_seq8.pdf, PDF file, 1.1 MB [file spectrum02093-21_supp_1_seq8.pdf]
